# RGB-D SLAM Using Point–Plane Constraints for Indoor Environments †

**DOI:** 10.3390/s19122721

**Published:** 2019-06-17

**Authors:** Ruibin Guo, Keju Peng, Weihong Fan, Yongping Zhai, Yunhui Liu

**Affiliations:** 1 College of Electronic Science and Technology, National University of Defense Technology, Changsha 410073, China; guoruibin08@nudt.edu.cn (R.G.); whfan@nudt.edu.cn (W.F.); 2 College of Advanced Interdisciplinary Studies, National University of Defense Technology, Changsha 410073, China; ypzhai@foxmail.com; 3 Department of Mechanical and Automation Engineering, Chinese University of Hong Kong, Hong Kong 999077, China; yunhui.liu@gmail.com

**Keywords:** visual SLAM, pose estimation, map reconstruction, point–plane-based factor graph, Manhattan World, RGB-D camera

## Abstract

Pose estimation and map reconstruction are basic requirements for robotic autonomous behavior. In this paper, we propose a point–plane-based method to simultaneously estimate the robot’s poses and reconstruct the current environment’s map using RGB-D cameras. First, we detect and track the point and plane features from color and depth images, and reliable constraints are obtained, even for low-texture scenes. Then, we construct cost functions from these features, and we utilize the plane’s minimal representation to minimize these functions for pose estimation and local map optimization. Furthermore, we extract the Manhattan World (MW) axes on the basis of the plane normals and vanishing directions of parallel lines for the MW scenes, and we add the MW constraint to the point–plane-based cost functions for more accurate pose estimation. The results of experiments on public RGB-D datasets demonstrate the robustness and accuracy of the proposed algorithm for pose estimation and map reconstruction, and we show its advantages compared with alternative methods.

## 1. Introduction

This article is an extension of a recent conference paper [1] that presented the exploitation of plane features to estimate sensors’ poses for low-texture indoor environments. Robust pose estimation and environment mapping are of great significance in the execution of robotic tasks, such as motion control and navigation. The robot’s pose and the scene’s map can be obtained by utilizing robotic sensors, such as wheel encoders, inertial measurement units [2,3,4], lasers [5,6], and cameras [7,8,9]. Among these solutions, the visual-based method is one of the more effective approaches because cameras can conveniently capture informative images to estimate the robot’s poses and perceive its surroundings. Although there have been plenty of methods using monocular, stereo, or RGB-D cameras for pose estimation and 3D mapping, daunting challenges remain for structural and low-texture environments for several reasons. For instance, in existing point-based methods [10,11], key steps in pose estimation, such as image aligning and computing the transformation matrix, heavily rely on feature points or high-contrast pixels. However, feature points are generally absent in structural and low-texture environments, and these methods can fail to estimate poses or result in low-accuracy estimations. To solve this problem, high-level features, such as lines and planes, are required.

Most indoor environments have many parallel and orthogonal lines and planes (called the Manhattan World [12]), and these high-level features can be exploited to improve the performance of pose estimation. Since these line and plane features can be easily calculated by using RGB-D cameras, which provide both depth information and a color image, the RGB-D camera has become a popular alternative to monocular and stereo cameras for the purpose of simultaneous localization and mapping (SLAM) tasks in indoor environments. These structural regularities have been exploited in studies [13,14,15,16] to estimate drift-free rotation, and by decoupling the rotation and translation components, pose estimation accuracy and map quality have been markedly improved by using the Manhattan World (MW) assumption with an RGB-D camera in scenes that satisfy the MW assumption.

In this paper, we propose a robust and accurate approach to pose estimation and 3D mapping using an RGB-D camera. We detected and matched point features from the color images by using the oriented fast and rotated brief (ORB) descriptor, and we detected and tracked multiple planes from the depth images on the basis of a motion model. Then, we exploited these features to construct cost functions to solve the pose of each captured frame and point–plane landmarks in local and global maps. Meanwhile, we added an orientation constraint to the loop detection process to reduce the drift error and avoid mismatches using an appearance constraint. Furthermore, we extracted the MW axes in the first captured frame of MW scenes, and we added the MW constraint to the previous point–plane-based cost functions to improve their performance.

Our algorithm exploits point and plane features and adds the MW constraint for pose estimation and scene reconstruction, which can perform well in harsh environments with low texture as well as general indoor environments. The contributions of this work are as follows:

- We exploited point and plane features, which provide reliable constraints for the estimation of poses and reconstruction of the scene’s map for the majority of indoor environments.
- We added the MW constraint to point–plane-based cost functions, resulting in the provision of fixed-plane normals as global landmarks for more accurate pose estimation.
- We evaluated our proposed approach on two public available datasets, and we obtained robust and accurate performance.

## 2. Related Work

The existing RGB-D SLAM methods for structural and low-texture environments can be divided into three classes: plane-based methods, dense methods, and MW-based methods.

Plane-based methods use plane features to construct and solve the optimization function for pose estimation. Lee et al. [17] presented a fast plane extraction and matching method for indoor mapping, and Taguchi et al. [18] used both points and planes as primitives to realize the registration of different 3D data. Khoshelham et al. [19] proposed a no-iteration pose estimation method based on point–plane correspondences. Thomas et al. [20] presented a structured 3D representation with a point-to-plane relationship to correct the deformations, and local and global mapping were processed to reduce the accumulation error and obtain an accurate large-scale 3D model. Kaess [21] presented a minimal representation for planar features and introduced a relative plane formulation that improved the convergence properties for faster pose optimization. The plane-based methods mentioned above require plane extraction and matching for each frame to construct the optimization function. Since there are no plane descriptors to perform plane matching, it is achieved by utilizing additional odometry sensors, such as wheel encoders or an inertial measurement unit. However, these additional sensors increase the complexity of the SLAM system and may not be available in some circumstances, so plane matching methods that use only the RGB-D frame need to be developed.

In dense methods, almost all pixels are used to estimate the pose. Newcombe et al. [22] presented a frame-to-global method that maintained the single-scene model with a global volumetric so that each new frame would be integrated into the volumetric. Whelan et al. [23] used a rolling cyclical buffer to operate in large environments and used place recognition for loop closing. Kerl et al. [24] proposed a dense visual SLAM method for RGB-D cameras that minimized both the photometric and depth error of all pixels, and Prisacariu et al. [25] presented a robust dense RGB-D SLAM method (InfiniTAM) that had low computational cost with RGB and depth constraints. These dense methods solve the pose with a dense vision front-end and are robust in low-texture environments. However, the number of points processed for each frame is large (typically hundreds of thousands), which makes the optimization computationally infeasible in real time without GPU implementation.

MW-based methods estimate the pose by decoupling the rotation and translation components. These methods utilize line and plane features to achieve a drift-free rotation matrix with the MW constraint, and the translational accuracy can be improved by using drift-free rotation. Zhou et al. [26] developed a mean shift paradigm to extract and track planar modes to achieve drift-free rotation, and they estimated the translation using three simple 1D density alignments in man-made environments. In the work of Kim et al. [27], lines and planes were exploited to estimate drift-free rotation, and the translation was recovered by minimizing the de-rotated reprojection error. Kim et al. [28] also proposed a linear SLAM method based on the Bayesian filtering framework for MW scenes. These methods have produced good SLAM performance results in MW scenes, but if the MW assumption is invalid, MW-based methods fail to estimate the pose or reconstruct the map.

## 3. Proposed Method

**Figure 1:**
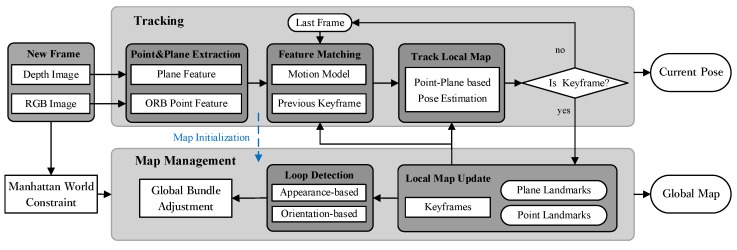
Overview of our point–plane-based RGB-D SLAM system. The inputs of the system are the RGB and depth images, and it can output the camera pose and scene’s map. We estimate the camera poses by using the tracked point and plane features. We exploit the point and plane constraints to update the local and global maps. The MW constraint is added to the map management part if the global MW axes are extracted from the first captured RGB-D frame.

We propose a point–plane-based RGB-D SLAM system that exploits point and plane features to estimate the camera pose and generate the 3D global map for indoor environments. Our proposed system has two main parts: (1) we detect and track the point and plane features with respect to the local map for each new captured frame, and we estimate the current pose by solving the cost function that is constituted by the tracked features (tracking part); and (2) we update the local map that consists of point–plane landmarks and keyframes for each new inserted keyframe, and we process the full bundle adjustment to obtain the global map if a loop is detected (map management part). If the current environment satisfies the MW assumption, we add the MW direction to constrain the normal of plane landmarks for more accurate pose estimation. The overview of our proposed system is shown in Figure 1.

### 3.1. Preliminaries

In this section, we first introduce the representations for the point and plane features that are extracted from the color and depth images, respectively. Then, we detail the state transformation and distance measurement, which are essential for constructing the cost functions and solving the nonlinear graph optimization problem for pose estimation.

#### 3.1.1. Point and Plane Representation

We extract ORB features for point tracking, as these features are computed extremely quickly, and they present good invariance to the camera’s auto-gain, auto-exposure, and changes in illumination. The point feature’s 2D pixel coordinate in the color image domain is defined as $u^{c}={\left(u^{c},v^{c}\right)}^{T}$, where c represents the current processing frame, and *u* and *v* represent the coordinate values on the *x*-axis and *y*-axis, respectively. For the aligned color and depth images, the point feature’s 2D coordinates are the same in the depth image and color image, so the corresponding value of $u^{c}$ in the depth image is represented by $d(u^{c})$. The point feature’s 3D position $X^{c}$ is reconstructed by using the inverse projection function $P^{-1}\left(\cdot \right)$:

(1) $$X^{c}=P^{-1}\left(u^{c},d\left(u^{c}\right)\right)=d\left(u^{c}\right){\left(\frac{u^{c}-c_{x}}{f_{x}},\frac{v^{c}-c_{y}}{f_{y}},1\right)}^{T}\in R^{3}$$

where $f_{x}$ and $f_{y}$ are the focal lengths on the *x*-axis and *y*-axis, and ${\left(c_{x},c_{y}\right)}^{T}$ is the camera’s central coordinate.

We detect the planes from the depth images using a fast plane extraction algorithm [29], which has three steps: generate initial blocks, merge the blocks on the basis of agglomerative clustering, and refine the border pixels. With this approach, we can obtain the plane: $n^{T}\cdot X^{p}=d$, where $n={\left(n_{x},n_{y},n_{z}\right)}^{T}$ represents the unit normal vector of the plane, $X^{p}$ represents the 3D point lying on this plane, and *d* is the distance to the origin of the camera coordinate system. The plane can also be represented by a homogeneous vector, $\pi ={\left(\pi_{x},\pi_{y},\pi_{z},\pi_{w}\right)}^{T}$:

(2) $$\pi =Q\left(n,d\right)=\frac{1}{\sqrt{n_{x}^{2}+n_{y}^{2}+n_{z}^{2}+d^{2}}}\left[\begin{array}{c} n\\ -d \end{array}\right]\in S^{3}$$

where Q(·) is the normalized transfer function for a 4-dimensional vector, $S^{3}$ represents the unit sphere, which can be identified with a set of unit quaternions, so the operations on the quaternions are suited to the plane’s homogeneous representation [21].

#### 3.1.2. State Transformation

The 3D point $X^{c}$ in the current frame is transformed to $X^{w}=R_{w,c}\cdot X^{c}+t_{w,c}$ in the global coordinate, where $R_{w,c}\in SO\left(3\right)$ is the rotation matrix and $t_{w,c}\in R^{3}$ is the translation vector. When we use the rigid-body transformation matrix that defined as $T_{w,c}=\left[\begin{array}{cc} R_{w,c} & t_{w,c}\\ 0_{1\times 3} & 1 \end{array}\right]$, the transformation relationship is expressed as $\left[\begin{array}{c} X^{w}\\ 1 \end{array}\right]=T_{w,c}\cdot \left[\begin{array}{c} X^{c}\\ 1 \end{array}\right]$.

The plane $\pi^{c}$ in the current coordinate is transformed to the global coordinate by $\pi^{w}=Q\left(T_{w,c}^{-T}\cdot \pi^{c}\right)$, where $T_{w,c}^{-T}=\left[\begin{array}{cc} R_{w,c} & 0_{3\times 1}\\ -t_{w,c}^{T}\cdot R_{w,c} & 1 \end{array}\right]$. In terms of the plane’s normal-distance representation, its state transformation is represented by $\left[\begin{array}{c} n^{w}\\ -d^{w} \end{array}\right]=T_{w,c}^{-T}\cdot \left[\begin{array}{c} n^{c}\\ -d^{c} \end{array}\right]$.

#### 3.1.3. Distance Measurement

In the graph-based pose estimation problem, the error function is constructed from an edge that connects multiple nodes [30]. As the binary edge only connects two nodes, the error function measures the distance between these two nodes. For two 3D points $X_{1}$ and $X_{2}$, we use the 2-norm function ${∥\cdot ∥}_{2}$ to define their relative distance: $e^{X}={∥X_{1}-X_{2}∥}_{2}$.

For two planes $\pi_{1}$ and $\pi_{2}$, their relative distance is defined as $e^{\pi }={∥\log\left(\pi_{1}^{-1}\cdot \pi_{2}\right)∥}_{2}$, where $\pi^{-1}={\left(-\pi_{x},-\pi_{y},-\pi_{z},\pi_{w}\right)}^{T}$, and we use the quaternion multiplication to operate the planes’ multiplication:

(3) $$\pi_{1}^{-1}\cdot \pi_{2}=\left[\begin{array}{c} -\pi_{1x}\pi_{2w}-\pi_{1y}\pi_{2z}+\pi_{1z}\pi_{2y}+\pi_{1w}\pi_{2x}\\ \pi_{1x}\pi_{2z}-\pi_{1y}\pi_{2w}-\pi_{1z}\pi_{2x}+\pi_{1w}\pi_{2y}\\ -\pi_{1x}\pi_{2y}+\pi_{1y}\pi_{2x}-\pi_{1z}\pi_{2w}+\pi_{1w}\pi_{2z}\\ \pi_{1x}\pi_{2x}+\pi_{1y}\pi_{2y}+\pi_{1z}\pi_{2z}+\pi_{1w}\pi_{2w} \end{array}\right]$$

The function log(·) maps an element of $S^{3}$ to the 3D rotation vector:

(4) $$log\left(\pi \right)=\frac{2\cos^{-1}\left(\pi_{w}\right)}{\sqrt{\pi_{x}^{2}+\pi_{y}^{2}+\pi_{z}^{2}}}\left[\begin{array}{c} \pi_{x}\\ \pi_{y}\\ \pi_{z} \end{array}\right]$$

### 3.2. Pose Estimation with Point and Plane Features

We match the points and planes detected in the current frame with the point and plane landmarks in the local map, and we utilize the tracked point and plane features to construct a cost function for estimating the current pose. By combining the point and plane constraints, we can accurately estimate the pose in scenes, even those with less texture.

#### 3.2.1. Point and Plane Feature Tracking

As mentioned in Section 3.1.1, we extract the ORB point features and plane features from the current frame $F^{c}$. For the point features, we get the initial point matches between the current frame and the last reference keyframe by using the ORB descriptors. Then, we project the corresponding map points onto the current frame and discard some outlier matches on the basis of the projection error. The set of optimized point matches is defined as $X=\{\left(X_{i}^{c},X_{i}^{L}\right),i=1,2,\ldots m\}$, where $X^{c}$ represents the point feature’s 3D position in the current frame coordinate, and $X^{L}$ represents the 3D position of the point landmark in the local map.

Since there are no plane descriptors to perform plane matching, we search for plane matches by the motion-model-based distance constraint. If the previous two frames $F^{c-2}$ and $F^{c-1}$ were tracked successfully, we use the constant velocity motion model [10] to predict the current pose: $T_{w,c}^{predict}=T_{c-1,c-2}\cdot T_{w,c-1}$, where $T_{c-1,c-2}$ is the relative pose from $F^{c-2}$ to $F^{c-1}$, and $T_{w,c-1}$ represents the estimated pose for $F^{c-1}$. One detected plane $\left[\begin{array}{c} n^{c}\\ -d^{c} \end{array}\right]$ is transformed to $\left[\begin{array}{c} n_{predict}^{c}\\ -d_{predict}^{c} \end{array}\right]=T_{w,c}^{predict}\cdot \left[\begin{array}{c} n^{c}\\ -d^{c} \end{array}\right]$, and we obtain the plane matches when they meet:

(5) $$\begin{array}{c} \left\{\begin{array}{c} ∥{\left(n_{predict}^{c}\right)}^{T}\cdot n^{L}{∥}_{2}>0.95\\ |d_{predict}^{c}-d^{L}|<0.05 \end{array}\right. \end{array}$$

where $\left(n^{L},d^{L}\right)$ is the normal-distance representation of the plane landmark in the local map. The set of plane matches is defined as $P=\{\left(\pi_{i}^{c},\pi_{i}^{L}\right),i=1,2,\ldots n\}$, where $\pi^{c}$ represents the plane’s homogeneous representation extracted from the current frame, and $\pi^{L}$ represents the plane landmark in the local map.

To avoid incorrect plane matches in cluttered environments, we only select extracted planes that have enough points (more than 5000) lying on them to match with the plane landmarks in the local map. For parallel planes that satisfy the previous condition, we select the plane with the largest number of on-plane points. In this way, the number of incorrect matches can be effectively reduced.

#### 3.2.2. Robust Pose Estimation

We jointly utilize the tracked points and planes to construct a cost function for the current pose estimation. Pose $T_{w,c}$ can be computed by solving

(6) $$\left\{R_{w,c},t_{w,c}\right\}=\underset{R_{w,c},t_{w,c}}{\arg\min}(\sum_{i\in X}\rho (∥R_{w,c}\cdot X_{i}^{c}+t_{w,c}-X_{i}^{L}{∥}_{2}^{2})+\sum_{j\in P}\lambda_{j}∥\log\left(Q{\left(T_{w,c}^{-T}\cdot \pi_{j}^{c}\right)}^{-1}\cdot \pi_{j}^{L}\right){∥}_{2}^{2})$$

where ρ(·) is the robust Huber cost function, $\lambda_{j}$ represents the number of pixels in the *j*th tracked plane, and 3D point $X_{i}^{c}=P^{-1}\left(u_{i}^{c},d\left(u_{i}^{c}\right)\right)$.

The cost function (6) contains two parts that correspond to point and plane constraints. The accurate pose can be solved by minimizing Equation (6), even in a texture-less environment in which few points tracked. The point constraints ensure that the pose estimation is reliable even in scenes in which there are not enough planes to be visible.

**Keyframe Selection:** By the previous process, we always know the number of tracked points and planes for each frame. If there is only one tracked plane but the number of detected planes is larger than 2 or the number of the tracked points is less than a threshold, this frame is selected as a keyframe. By inserting the keyframes and updating the local map, the drift error of the pose estimation can be markedly reduced.

### 3.3. Map Management and Loop Detection

In this section, we describe the operation for the local map when a new keyframe is inserted, and we detect the closing loop on the basis of both the appearance and orientation constraints. If loop detection is successful, the full bundle adjustment is performed to generate the final global map.

Similar to the co-visibility graph and essential graph in ORB-SLAM [10], we denote the set of co-visible keyframes by $K^{L}$; all points seen in $K^{L}$ are represented by $S^{L1}$, and all planes seen in $K^{L}$ are represented by $S^{L2}$. All other keyframes $K^{F_{1}}$ that are not in $K^{L}$, as well as the observation points in $S^{L1}$, contribute to the cost function but remain fixed in the optimization. All other keyframes $K^{F_{2}}$ that are not in $K^{L}$, as well as the observation planes in $S^{L2}$, contribute to the cost function and also remain fixed in the optimization. We define the set of point matches as $X^{L_{m}}$ between the points in $S^{L1}$ and keypoints in keyframe *m*, and we define the set of plane matches as $P^{L_{n}}$ between the planes in $S^{L2}$ and the keyplanes in keyframe *n*. The local map is updated by solving

(7) $$\begin{array}{cc} \left\{X_{i}^{L},\pi_{j}^{L},R_{w,l}^{k},t_{w,l}^{k}|i\in S^{L1},j\in S^{L2},l\in K^{L}\right\} & =\underset{X_{i}^{L},\pi_{j}^{L},R_{w,l}^{k},t_{w,l}^{k}}{\arg\min}\left(\sum_{m\in K^{L\cup F_{1}}}\sum_{p\in X^{L_{m}}}\rho \left(E_{mp}^{X_{L}}\right)+\sum_{n\in K^{L\cup F_{2}}}\sum_{q\in P^{L_{n}}}\lambda_{q}\cdot E_{nq}^{\pi_{L}}\right)\\ E_{mp}^{X_{L}} & =∥R_{w,m}^{k}\cdot X_{p}^{m}+t_{w,m}^{k}-X_{p}^{L}{∥}_{2}^{2}\\ E_{nq}^{\pi_{L}} & =∥\log\left(Q{\left(T_{w,n}^{-T}\cdot \pi_{q}^{n}\right)}^{-1}\cdot \pi_{q}^{L}\right){∥}_{2}^{2} \end{array}$$

where $\lambda_{q}$ represents the number of pixels contained in the plane.

#### 3.3.1. Local Map Update

**Figure 2:**
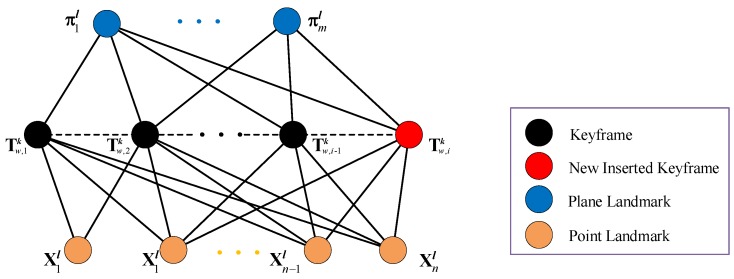
Point–plane-based factor graph that is used to represent the local map. Four colors denote four different elements. When a new keyframe (red circle) is inserted, the pose of the keyframes (black circles), point landmarks (blue circles), and plane landmarks (yellow circles) are optimized by minimizing the error cost function constructed by the factor graph.

The local map contains three elements: the keyframe, point landmark, and plane landmark. These elements are represented by nodes in a factor graph, which is shown in Figure 2. There are two kinds of binary edges in this point–plane-based factor graph: one connects the keyframe node and point landmark node, and the other one connects the keyframe node and plane landmark node. When a new keyframe is inserted, the poses of all elements in the local map are optimized by the local bundle adjustment (BA).

#### 3.3.2. Loop Detection based on Appearance and Orientation Constraints

**Figure 3:**
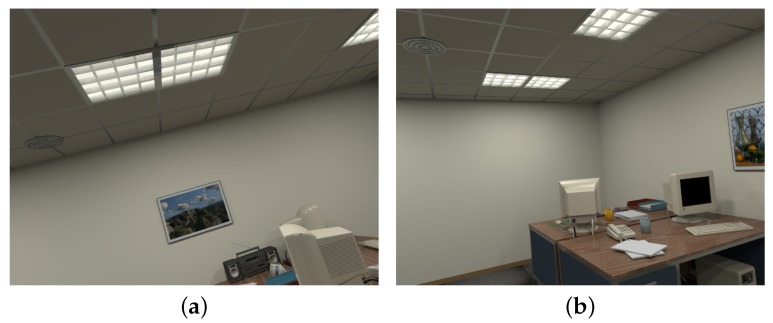
Two mismatched loop images based on the appearance constraint: (**a**) Color image of Frame 182 in the ‘OfficeRoom1’ sequence of the ICL-NUIM dataset. (**b**) Color image of Frame 319 in the ‘OfficeRoom1’ sequence. These two images are viewed from two completely different perspectives, but their similar appearance score is high because the ceiling is a common feature in the environment.

In the point-based SLAM system, loop detection is performed by using a bag-of-words (BoW) place recognition module with DBoW [31]. This visual vocabulary is an appearance constraint for loop detection, and it is created by pretraining with a large set of pictures. Since places that appear similar in indoor environments are familiar, as shown in Figure 3, we add an orientation constraint to complement the appearance constraint in determining the loop keyframes.

In our proposed system, if the similarity score (using the visual vocabulary) between a new inserted keyframe $K_{i}$ and an existing keyframe $K_{p}^{L}$ is higher than a threshold, we select this keyframe as the potential loop keyframe, and we compute their orientation distance in degrees by Equation (8). We confirm loop detection if the potential loop keyframe meets $d_{p,i}^{O}<90deg$. By adding this orientation constraint, the mismatch (based on the appearance constraint) of these two loop images is revised.

(8) $$d_{p,i}^{O}=\arccos\left(\frac{tr(R_{w,p}^{T}\cdot R_{w,i})-1}{2}\right)\times 57.3$$

where tr(·) denotes the trace of a matrix, and $R_{w,i}$ and $R_{w,p}$ represent the rotation matrix component for keyframe $K_{i}$ and $K_{p}^{L}$, respectively.

#### 3.3.3. Global Map Generation

If a loop is detected in the previous step, all poses of the elements are optimized by the full bundle adjustment, and the first keyframe is fixed in the process of full BA. We represent the set of all keyframes by $K^{G}$: all point landmarks are represented by $S^{G1}$, and all plane landmarks are represented by $S^{G2}$. We define the set of point matches as $X^{G_{m}}$ between points in $S^{G1}$ and keypoints in keyframe *m*, and we define the set of plane matches as $P^{G_{n}}$ between planes in $S^{G2}$ and planes in keyframe *n*. The global map is optimized by solving

(9) $$\begin{array}{cc} \left\{X_{i}^{G},\pi_{j}^{G},R_{w,l}^{k},t_{w,l}^{k}|i\in S^{G1},j\in S^{G2},l\in K^{G}\right\} & =\underset{X_{i}^{G},\pi_{j}^{G},R_{w,l}^{k},t_{w,l}^{k}}{\arg\min}\left(\sum_{m\in K^{G}}\sum_{p\in X^{G_{m}}}\rho \left(E_{mp}^{X_{G}}\right)+\sum_{n\in K^{G}}\sum_{q\in P^{G_{n}}}\lambda_{q}\cdot E_{nq}^{\pi_{G}}\right)\\ E_{mp}^{X_{G}} & =∥R_{w,m}^{k}\cdot X_{p}^{m}+t_{w,m}^{k}-X_{p}^{G}{∥}_{2}^{2}\\ E_{nq}^{\pi_{G}} & =∥\log\left(Q{\left(T_{w,n}^{-T}\cdot \pi_{q}^{n}\right)}^{-1}\cdot \pi_{q}^{G}\right){∥}_{2}^{2} \end{array}$$

where $\lambda_{q}$ represents the number of pixels in the plane.

After the full BA, the global map with point and plane landmarks is generated, which can be applied to robotic localization, navigation, and path planning.

### 3.4. Pose and Plane Optimization with the MW Constraint

For the environment that satisfies the MW assumption, we exploit the parallel and orthogonal lines and planes to extract the MW axes, and we add the MW constraint to construct the cost function to optimize the poses of keyframes and landmarks.

#### 3.4.1. MW Axes Extraction

**Figure 4:**
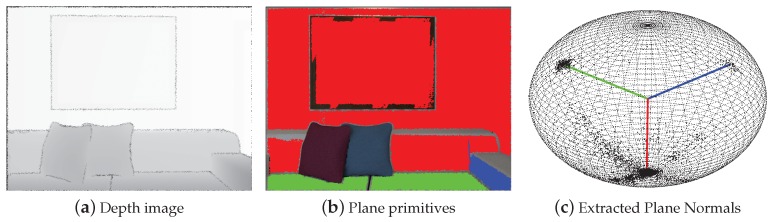
Result of the plane normal extraction: (**a**) depth image of Frame 0 in the ‘Living Room 1’ sequence of the ICL-NUIM dataset; (**b**) detected planes using fast plane extraction method; (**c**) extracted plane normals on the unit sphere. The plane primitives in the image domain and their corresponding extracted normal vectors are in the same color.

We extract the MW axes from the first frame by utilizing the plane normal vectors and the parallel lines’ vanishing directions (VDs), the details of which are given in our previous work [32]. To extract the accurate plane normals, we use the normal vectors obtained by the previous fast plane extraction method as the initial value and then perform the mean shift algorithm in the tangent plane of the unit sphere to get the final plane normal vectors, as shown in Figure 4. As the normals of parallel planes are regularly distributed and more likely to be around the ground MW axes on the unit sphere, the final extracted results are obtained by utilizing all normals of parallel planes, which are more accurate than the initial plane normals.

**Figure 5:**
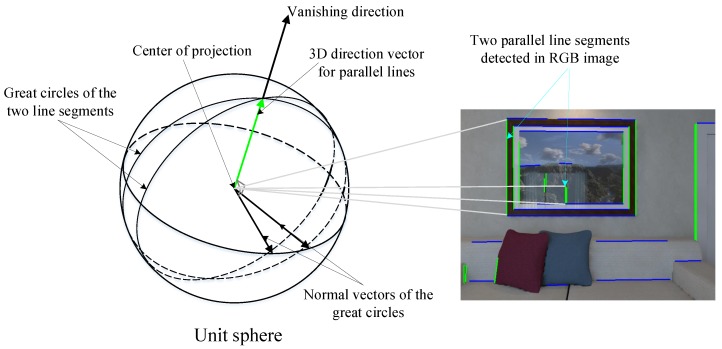
Three-dimensional geometric relationship between parallel lines and their vanishing direction. The unit sphere is in the center of a camera projection. Two parallel lines are projected onto the unit sphere as two great circles, and the vanishing direction is obtained by the cross-projection of these two great circles’ normal vectors. Two parallel lines and their corresponding vanishing direction are drawn in red.

The geometric relationship between the VDs and parallel lines is shown in Figure 5. To extract accurate VDs ($d_{k}^{v},k=1,2,3$), we use the simplified Expectation–Maximization (EM) clustering method to group image lines and compute their corresponding 3D direction vectors. We use the linear-time Line Segment Detector (LSD) [33] to extract 2D line segments from the color image and roughly cluster the lines using the K-means method. Then, we compute the VDs by solving the weighted objective function for each $d_{k}^{v}$:

(10) $$d_{k}^{v}=argmin\sum_{i}\frac{length(l_{i}^{\left(k\right)})}{max\left(length\left(l^{\left(k\right)}\right)\right)}\cdot {\left(l_{i}^{{}^{(k)T}}Kd_{k}^{v}\right)}^{2}$$

where $l^{\left(k\right)}$ represents the *i*th line cluster obtained by the K-means method, $length(l_{i}^{\left(k\right)})$ represents the length of the *i*th line, $max\left(length\left(l^{\left(k\right)}\right)\right)$ represents the maximum line length in cluster *k*, and K represents the internal camera parameters.

**MW Axes Seeking:** In the scenes that satisfy the MW assumption, there are three fixed axes $\left(r_{1}^{g}\;\;r_{2}^{g}\;\;r_{3}^{g}\right)$. It should be noted that we treat $r^{g}$ and $-r^{g}$ as the same direction. To determine the MW axes, we first get a redundant set by using the plane normals and VDs obtained using the previous method. Then, we seek the plane that contains the most pixels and set its plane normal as the first MW axis $r_{1}$. The other two MW axes $r_{2}$ and $r_{3}$ are sought on the basis of two principles: the number of pixels belonging to the plane or line and the orthogonal constraint. The larger the number of pixels, the higher the priority of the plane normal or VD. The final global MW axes are obtained by using singular value decomposition (SVD):

(11) $$\left[r_{1}^{g}\;\;r_{2}^{g}\;\;r_{3}^{g}\right]=UV^{T}$$

where $\left[U,D,V\right]=SVD\left(\left[\lambda_{1}r_{1}\;\;\lambda_{2}r_{2}\;\;\lambda_{3}r_{3}\right]\right)$, and factor $\lambda_{i}$ represents the number of pixels belonging to a plane or line.

#### 3.4.2. Optimization with Fixed Plane Normal

In the previous section, we present the extraction of the global MW axes $\left(r_{1}^{g}\;\;r_{2}^{g}\;\;r_{3}^{g}\right)$, which are used to fix the normals of plane landmarks during optimization. We add the MW constraint to construct the cost functions (6), (7) and (9), in which the plane landmarks are represented by

(12) $$\pi_{i}^{L_{fixed}}=Q\left(r_{i}^{g},d_{i}\right)=\frac{1}{\sqrt{r_{ix}^{2}+r_{iy}^{2}+r_{iz}^{2}+d_{i}^{2}}}\left[\begin{array}{c} r_{i}^{g}\\ -d_{i} \end{array}\right]\in S^{3}$$

For MW environments, this MW constraint can effectively improve the accuracy of the SLAM system because adding the MW axes to the cost function is equivalent to setting three global directions in the optimization for poses and landmarks, so the drift can be reduced. If the MW axes are not detected in the current environment, we simply use the point–plane-based SLAM without the MW constraint to perform the localization and mapping tasks.

## 4. Results

We evaluated our proposed approach on a synthetic dataset (ICL-NUIM [34]) and a real-world dataset (TUM RGB-D [35]). All experiments were run on a desktop computer with an Intel Core i7, 16 GB memory, and Ubuntu 16.04 platform. Our proposed system was built on ORB-SLAM2 [10], and our system is executed in the same manner as ORB-SLAM2.

- The ICL-NUIMdataset comprises images from a hand-held RGB-D camera in synthetically generated environments. These sequences were captured in a living room and an office with perfect ground-truth poses to fully quantify the accuracy of a given visual odometry or SLAM system. Depth and RGB noise models were used to alter the ground images to simulate realistic sensor noise. Some image sequences are in low-texture environments, which makes it difficult to estimate the poses of the whole images in these sequences.
- The TUM RGB-D dataset is a famous benchmark that is used to evaluate the accuracy of a given visual odometry or visual SLAM system. It contains various indoor sequences captured by a Kinect RGB-D sensor. The sequences were recorded in real environments at a frame rate of 30 Hz with a 640×480 resolution, and their ground-truth trajectories were obtained from a high-accuracy motion-capture system. The TUM dataset is more challenging than the ICL dataset because includes some blurred images and inaccurate alignment image pairs that make it difficult to estimate the camera poses.

We compared our proposed approach with five methods: ORB-SLAM2 [10], DVO [24], InfiniTAM [25], LPVO [27], and L-SLAM [28]. ORB-SLAM2 is a state-of-the-art point-based SLAM system; DVO estimates the robust poses with photometric and depth error by using the color and depth images together; InfiniTAM estimates the camera poses from the RGB and depth images with a GPU in real time; LPVO exploits the line and plane to estimate the zero-drift rotation and then estimates the 3D poses with tracked points in the MW scenes; L-SLAM estimates the camera position and plane landmarks with a linear SLAM formulation in the MW environments. We use the root-mean-square error (RMSE) of the absolute translational error (ATE) as the performance metric for the entire sequences:

(13) $$\mathrm{ATE}.RMSE=\frac{1}{N}\sum_{i=1}^{N}{∥R_{g,p}\cdot X_{i}^{p}+t_{g,p}-X_{i}^{g}∥}_{2}$$

where $R_{g,p}$ and $t_{g,p}$ represent the rotation matrix and translational matrix that transform the trajectory coordinate obtained by our proposed method to the ground-truth coordinate; three-dimensional points $X_{i}^{p}$ and $X_{i}^{g}$ denote the traces of the proposed method and ground truth, respectively; and *N* represents the number of frames in the tested sequence.

### 4.1. Evaluation on Synthetic Dataset

**Figure 6:**
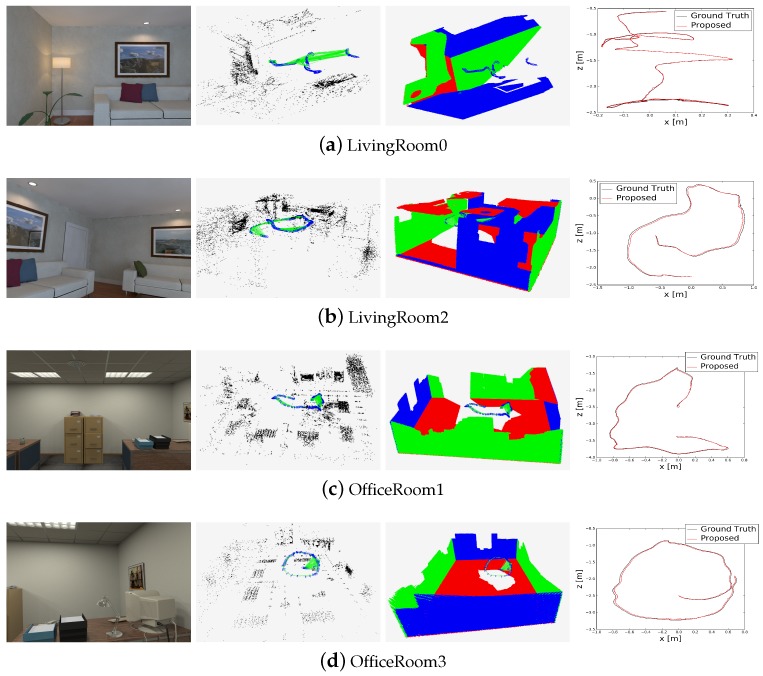
Results of camera poses and landmarks estimated by our proposed method on the ICL-NUIM dataset: (**a**) ‘Living Room 0’; (**b**) ‘Living Room 2’; (**c**) ‘Office Room 1’; (**d**) ‘Office Room 3’. For each sequence, the four images from left to right represent, respectively, one color image in the current sequence, the point landmarks (black dots) obtained by our proposed method, the plane landmarks obtained by our proposed method, and the trajectory comparison between the ground truth and our proposed method. The estimated keyframe trace (blue boxes) and connection graph between them (green lines) were added to the middle images that show the point and plane landmarks.

We first evaluated our proposed method on the ICL-NUIM dataset. The estimated trajectories and point–plane landmarks are shown in Figure 6, and the measured RMSE values of the ATE for each sequence are shown in Table 1. The smallest values are bolded and indicate the most accurate result for the pose estimation. For example, in ‘Living Room 0’, the ATE.RMSE value of our proposed method is 0.006 m, while those of ORB-SLAM2, DVO, LPVO, and L-SLAM are 0.010, 0.108, 0.015, and 0.012 m, respectively. The ‘Living Room 1’ sequence includes images that are mostly composed of a texture-less wall, so the accuracy of the point-based ORB-SLAM2 method is poor. As the DVO method does not have an efficient loop-closing process, the drift error cannot be avoided, and its ATE.RMSE is large. InfiniTAM fails to estimate the whole frames’ poses in three sequences (“Living Room 0”, “Living Room 3”, and “Office Room 2”) because there are some frames with only one visible plane in the depth image and low texture in the color image. We marked the result as ‘×’. Although the LPVO method can provide drift-free rotation, it estimates the 3D pose using only the tracked points; thus, if there are not enough points, the accuracy decreases. L-SLAM is a linear SLAM method that uses the MW constraint and does not need to estimate the 3 degrees of freedom rotation. L-SLAM performs well with the MW scenes, and our method is comparable to it. The last column in Table 1 shows the number of frames in the current sequence.

**Figure 7:**
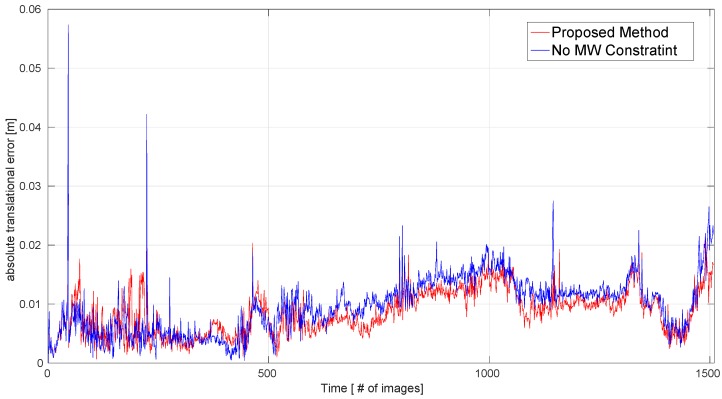
Performance evaluation for the MW constraint on the ‘Living Room 0’ sequence. Absolute translational errors for our proposed method with and without the MW constraint are compared.

The MW assumption is sufficiently suitable for the ICL-NUIM benchmark. To clearly show the effect of the MW constraint, we measured the ATE.RMSE for all sequences obtained by our method without the MW constraint; this corresponds to the ‘No MW’ column in Table 1. We recorded the values of the absolute translational error (ARE) for each frame in the ‘Living Room 0’ sequence, and the ATE values with and without the MW constraint are shown in Figure 7: in this figure, the smaller the ATE value, the more accurate the pose estimation. This demonstrates that the MW constraint can improve the accuracy of pose estimation for MW environments.

### 4.2. Evaluation on Real-World Data

**Figure 8:**
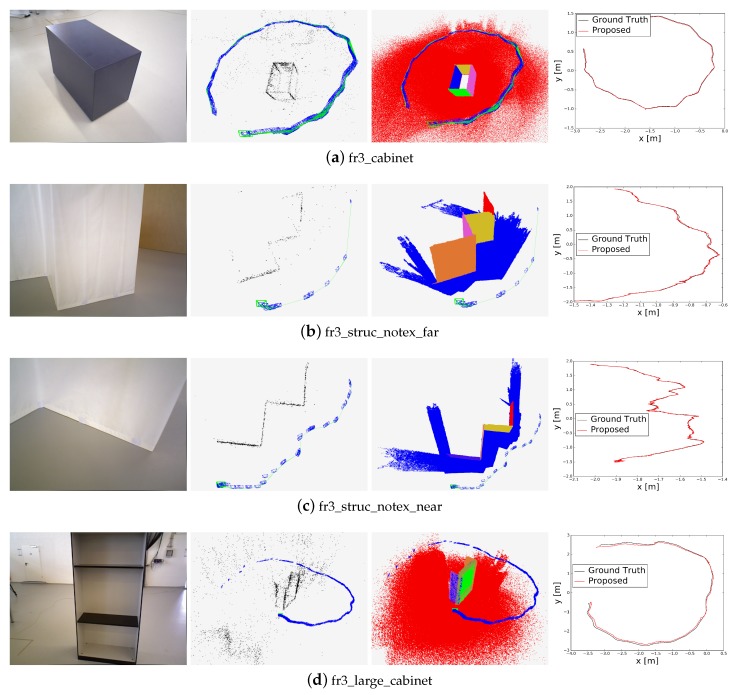
Results of camera poses and landmarks estimated by our proposed method on the TUM RGB-D dataset: (**a**) ‘fr3_cabinet’; (**b**) ‘fr3_struc_notex_far’; (**c**) ‘fr3_struc_notex_near’; (**d**) ‘fr3_large_cabinet’. For each sequence, four images from left to right represent, respectively, one color image in the current sequence, the point landmarks (black dots) obtained by our proposed method, the plane landmarks obtained by our proposed method, and the trajectory comparison between the ground truth and our proposed method. The estimated keyframe trace (blue boxes) and connection graph between them (green lines) were added to the middle images that show point and plane landmarks.

We then evaluated our proposed method on the TUM RGB-D dataset. The trajectories generated by the poses of the whole captured frames and the point–plane landmarks in the map are shown in Figure 8. We compared the performance of our proposed algorithm with that of the other five methods on six real-world TUM RGB-D sequences that contain structural regularities. The comparison results are shown in Table 2. We provide the ATE.RMSE for 3D pose estimation, and the smallest values are indicated in bold. Our proposed method performs better in low-texture environments because it uses the point and plane features to estimate poses. In ‘fr3_cabinet’, the ORB-SLAM2 method failed to estimate the poses for the entire sequence because there are not enough reliable tracked points for some frames; we marked the result as ‘×’ in Table 2. The last column in Table 2 also represents the number of frames in the current TUM RGB-D sequence.

**Figure 9:**
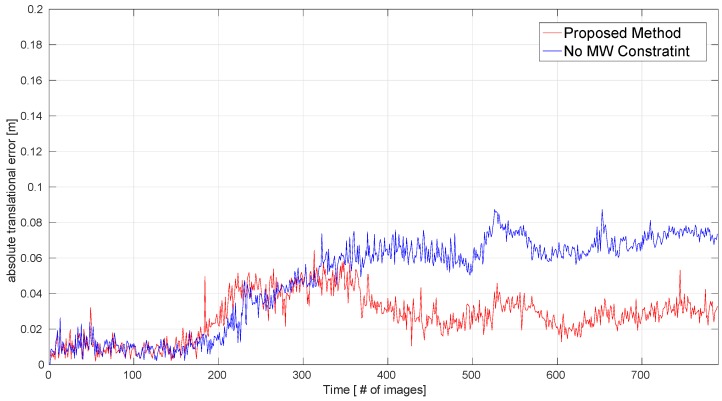
Performance evaluation for the MW constraint on the ’fr3_struc_notex_far’ sequence. Absolute translational errors for our proposed method with and without the MW constraint are compared.

The performance results of the MW constraint on the ‘fr3_struc_notex_far’ sequence is shown in Figure 9. The final translational drift obtained by our proposed method with and without the MW constraint is 0.031 and 0.072, respectively. It is clear that the MW constraint can effectively reduce the drift error for the MW scenes.

## 5. Conclusions

We proposed a point–plane-based method to estimate robot poses and reconstruct the maps of scenes of indoor environments using an RGB-D camera. We exploited point and plane features to generate reliable constraints, which we applied to the constructed cost function for solving the transformation matrix, and we used minimal representation for planes in the nonlinear optimization process. We developed a vanishing direction extraction method based on parallel lines and combined it with the detected plane normals to seek the MW axes in the current environment. Then, we added the MW constraint to further improve accuracy for MW environments. The proposed algorithm was tested on both synthetic and real-world publicly available RGB-D datasets, and we compared the pose estimation performance of our method with that of five existing methods. The results demonstrate the accuracy and robustness of the proposed method. Our approach can be used for a robot’s tasks in indoor environments. In future work, we will extend our approach to the point–line–plane feature fusion SLAM system, which may provide robust pose estimation in more general environments and generate structural maps.

## Figures and Tables

**Table 1:** Comparison of ATE.RMSE (unit: m) on the ICL-NUIM dataset. The smallest values are bolded, which indicates the most accurate method for the pose estimation.

Sequence	Proposed	No MW	ORB-SLAM2	DVO	InfiniTAM	LPVO	L-SLAM	Frames
Living Room 0	**0.006**	0.007	0.010	0.108	×	0.015	0.012	1508
Living Room 1	0.010	0.011	0.185	0.059	**0.006**	0.039	0.027	965
Living Room 2	0.026	0.027	0.028	0.375	**0.013**	0.034	0.053	880
Living Room 3	**0.013**	0.016	0.014	0.433	×	0.102	0.143	1240
Office Room 0	**0.019**	0.025	0.049	0.244	0.042	0.061	0.020	1507
Office Room 1	0.016	0.017	0.079	0.178	0.025	0.052	**0.015**	965
Office Room 2	**0.017**	0.019	0.025	0.099	×	0.039	0.026	880
Office Room 3	0.016	0.018	0.065	0.079	**0.010**	0.030	0.011	1240

**Table 2:** Comparison of ATE.RMSE (unit: m) on the TUM RGB-D Dataset. The smallest values are bolded, which indicates the most accurate method for the pose estimation.

Sequence	Proposed	No MW	ORB-SLAM2	DVO	InfiniTAM	LPVO	L-SLAM	Frames
fr3_struc_notex_far	**0.017**	0.029	0.276	0.213	0.037	0.075	0.141	790
fr3_struc_tex_far	**0.011**	0.012	0.024	0.048	0.030	0.174	0.212	904
fr3_struc_notex_near	**0.008**	0.009	0.652	0.076	0.022	0.080	0.066	1031
fr3_struc_tex_near	**0.011**	0.013	0.019	0.031	0.034	0.115	0.156	1054
fr3_cabinet	**0.012**	0.013	×	0.690	0.035	0.520	0.291	926
fr3_large_cabinet	**0.074**	0.094	0.179	0.979	0.512	0.279	0.140	979
